# Malnutrition-Related Factors Increased the Risk of Anastomotic Leak for Rectal Cancer Patients Undergoing Surgery

**DOI:** 10.1155/2020/5059670

**Published:** 2020-04-30

**Authors:** Hao Xu, Fanmin Kong

**Affiliations:** Department of Gastrointestinal Surgery, The First Hospital of China Medical University, Shenyang, China

## Abstract

**Objective:**

To study the possible risk factors and related prediction indexes of anastomotic leakage (AL) in patients with rectal cancer during the perioperative period and to provide effective indexes for predicting whether AL will occur in postoperative patients with rectal cancer and whether early nutritional support is needed.

**Background:**

AL after rectal cancer surgery is a common and serious complication. Many of the risk factors for AL have been confirmed. Nevertheless, the evidence of the effect of perioperative malnutrition on AL is still insufficient. This article will make a further study on this point.

**Methods:**

We collected perioperative clinical data from 382 patients with rectal cancer who underwent surgery from September 2015 to May 2017. After 1 month of follow-up, relevant risk factor data were collected and analyzed.

**Results:**

Data analysis showed that the incidence of AL was 14.65%. In single factor analysis, patients with high score of NRS-2002, high score of PG-SGA, diabetes, perioperative blood transfusion, postoperative diarrhea, later tumor stage, high score of ASA, low postoperative albumin, and rectal cancer patients with tumor close to the anus may led to AL. Multivariate analysis revealed that low postoperative albumin (*p* = 0.044), tumor close to the anus (*p* = 0.004), diabetes (*p* = 0.003), perioperative blood transfusion (*p* < 0.001), diarrhea (*p* = 0.005), later tumor stage, and high score of PG-SGA (*p* < 0.001) were the independent risk factors for postoperative AL.

**Conclusions:**

AL in rectal cancer operation is a common postoperative complication. Patients with diabetes or high PG-SGA score or low perioperative albumin will have increased risk factors of AL, which should be paid enough attention in the perioperative period and nutritional support should be provided as soon as possible. Patients who have incomplete intestinal obstruction but can make effective intestinal preparation or who receive neoadjuvant chemotherapy have no increased risk of AL.

## 1. Introduction

Because of the influence and limitations of various clinical factors, postoperative AL in rectal cancer is one of the more serious and common complications of rectal cancer. With the development of clinical technology as well as innovations of science and technology, the incidence of AL has decreased; however, worldwide, the incidence of postoperative AL remains at 0–36% [1–4]. If this complication occurs, the perioperative mortality rate due to this complication is about 5%–20% [4–7]. In addition, rectal AL can lead to prolonged hospitalization, increased costs of medical care, and substantial pain in patients. In particular, postoperative AL delays the optimal chemotherapy period, even leading to unsuccessful chemotherapy treatment after surgery, creating risk of recurrence or metastasis [8, 9]. However, in clinical work, surgeons can only judge whether there is AL, but the occurrence of AL cannot be accurately predicted during the perioperative period, so as to take intervention as soon as possible. There are many factors leading to AL in rectal cancer [9, 10], many of which have been clinically confirmed. However, whether nutritional indicators have an effect on AL remains controversial. At present, there are few studies regarding AL and its relation with factors; not only that, there are few clinical guidelines explaining perioperative clinical management and intervention methods for rectal cancer patients with poor nutritional status. Therefore, the purpose of this study was to explore whether perioperative period nutritional indicators and nutritional status of patients had an effect on postoperative AL in rectal cancer and its possible causes.

## 2. Aims and Methods

This study was a retrospective, observational, single-center study of the effect of perioperative nutritional indicators on the incidence of postoperative AL in patients with rectal cancer. The main purpose was to confirm whether various preoperative, intraoperative, and postoperative nutritional indicators and other related factors affect postoperative AL of patients with rectal cancer.

We collected clinical data from 382 patients with rectal cancer who underwent surgery between September 2015 and May 2017. The inclusion criteria were (1) primary rectal malignant tumor without metastasis or resectable metastasis; (2) the tumor distance to the anus is ≤15 cm; (3) tumor resection and intestinal anastomosis were performed at the same stage; and (4) the patients can defecate independently before operation and there is no complete intestinal obstruction. The exclusion criteria were (1) the patient underwent emergency surgery; (2) the patient received neoadjuvant radiotherapy before operation; (3) patients have a preventive stoma due to poor bowel preparation during surgery; and (4) age is less than 18 years old or older than 85 years old. The 382 patients enrolled were strictly followed up for various indicators and anastomotic healing from the day after surgery. According to the definition of AL in the surgical infection research group in the United Kingdom in 1991 [11], the following conditions suggested AL: (1) the presence of AL confirmed by imaging, (2) clinical observation of intestinal content exudation in drainage tube, (3) confirmation of endoscopic or digital rectal examination, and (4) AL confirmed by emergency surgery.

Excluding some incomplete information or lost follow-up data, the final data of 382 patients were completely collected, including some nutritional indicators before and after surgery and potential risk factors for AL. Nutritional indicators included BMI, NRS-2002 score, PG-SGA score, albumin, and hemoglobin levels before and 4 days after surgery. Common factors include age, history of smoking or drinking, diabetes, hypertension, coronary artery disease, and TNM staging of tumors (AJCC 8th Edition). Perioperative factors included preoperative bowel preparation, incomplete intestinal obstruction, perioperative blood transfusion, surgical approach, postoperative diarrhea, ASA score, and neoadjuvant chemotherapy. The patients were followed up for 1 month after the surgery day, following up the patients or their families by mobile phone and using the data of outpatient system to access whether these patients have tumor recurrence.

The final collected clinical data were analyzed by statistical software SPSS 18.0 (SPSS, Chicago, IL, USA). The counting data and grade data were analyzed by Pearson chi-square test, and the measurement data were analyzed by independent sample *t*-test or nonparametric rank sum test. Further, the statistically significant factors were analyzed by logistic regression. Kaplan-Meier curve was drawn to analyze the recurrence and prognosis of the patients and the definition *p* < 0.2 was statistically significant.

## 3. Results

After meeting the inclusion and exclusion criteria, a total of 382 cases of data were included in the study. From Table 1, we can see that 56 patients had AL, accounting for 14.65% of the total number of cases. Among them, 36 were female patients and 20 were male. There were no significant differences in terms of age, height, weight, or BMI.

In the univariate analysis (Table 2), the following factors were found to be associated with AL: high score on NRS-2002, high score on PG-SGA, diabetes, perioperative blood transfusion, postoperative diarrhea, later tumor stage, surgical approach, and ASA score. In Table 3, we can also conclude that rectal cancer patients with tumors closer to the anus are more likely to have AL.

As described in Table 4, statistically significant factors were brought into logistic regression model for multivariate analysis. Multivariate regression analysis showed that postoperative low albumin (*p* = 0.044), tumor close to the anus (*p* = 0.004), diabetes (*p* = 0.003), perioperative blood transfusion (*p* < 0.001), diarrhea (*p* = 0.005), later tumor stage, and high PG-SGA score (*p* < 0.001) were independent risk factors for AL after rectal cancer surgery.

**Figure 1:**
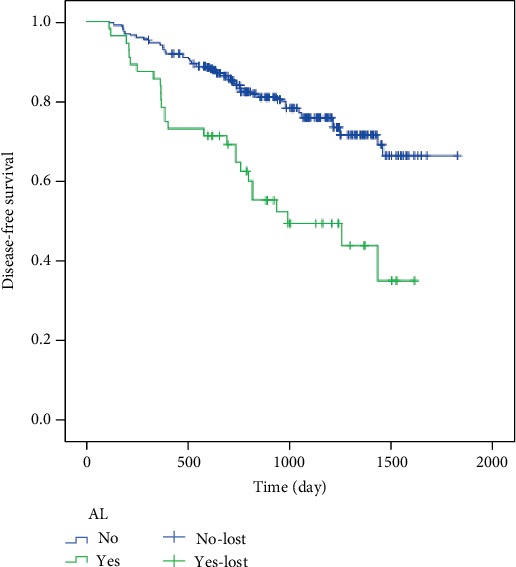
The DFS of the anastomotic leakage group was different from that of the nonanastomotic leakage group, which suggested that the patients of the anastomotic leakage group were more likely to have recurrence after operation.

Patients with complete intestinal obstruction are unable to make effective intestinal preparation, and patients who have received neoadjuvant radiotherapy did not join this study, because it is clear that the these factors will lead to AL. Prophylactic colostomy is usually chosen to ensure the safety of patients. Adding these data may lead to biased results. Analyzing from the results, neoadjuvant chemotherapy cannot increase the probability of AL, but the later tumor stage can increase the probability of AL. It may be due to the increase of the size of the tumor, which increases the difficulty of the operation, or the edema of the intestinal tract which leads to the poor healing condition of the anastomosis. In Figure 1, we can get that DFS in the AL group is different from that in the non-AL group, suggesting that the patients in the AL group are more likely to have recurrence after operation which the prognosis is poor. It is also consistent with the previous research results.

## 4. Discussion

In recent years, due to the development of imaging techniques such as CT and MRI, increasing numbers of asymptomatic or tiny AL have been discovered, making percutaneous drainage more common; advances in antibiotics have made common symptoms such as fever, tachycardia, and increase of infection index caused by AL to be no longer an important indication of reoperation. The most effective treatment in clinical practice is to choose surgical treatment such as enterostomy [23–25]. While there have been continuous improvements in examination methods, the incidence of postoperative AL in rectal cancer remains high in many large-sample studies, with fluctuations between 3% and 15.9% [12–14]. The incidence of AL in the rectum is higher, and the results of this study are also within this scope. Nevertheless, this study showed that the incidence of postoperative AL in rectal cancer patients with poor nutritional status remains high. This situation needs our more attention. Statistical analysis revealed a variety of factors, including low postoperative albumin, tumor close to the anus, diabetes, perioperative blood transfusion, diarrhea, later tumor stage, and high score of PG-SGA, which can lead to AL; these factors in the previous literature have also been confirmed. In addition, the study also found that in patients with incomplete intestinal obstruction, if there is adequate intestinal preparation, the incidence of anastomosis leakage does not seem to increase; neoadjuvant chemotherapy will not increase the incidence of AL as well. Unfortunately, due to sample size and single-center limitations, we can only find three independent risk factors related to nutritional status and AL in multivariate analysis. However, diabetes, high PG-SGA score, and low postoperative albumin are an imbalance of nutritional status. Therefore, nutritional assessment and replenishment of perioperative period patients are particularly important.

The nutritional status of patients with rectal cancer is an important factor leading to AL. It is also one of the hotspots for studying AL. A series of studies have reported associations of low-level serum albumin or low-level total protein with postoperative AL in rectal cancer [15–18]. Several studies have also shown that nutritional support for preoperative malnutrition patients reduced the incidence of AL and other complications [19, 20]. Therefore, nutritional support is recommended for patients with poor nutritional status prior to surgery. These nutritional supports are based on enteral nutrition, in order to reduce AL and other complications.

If postoperative AL occurs after surgery, early intervention is the guarantee for reducing mortality. Evidence for the diagnosis of AL after rectal cancer currently includes imaging examination, clinical presentations, and blood routine and biochemical examination. Imaging and clinical manifestations always tend to be lagging indicators. Recent studies have shown that CRP (C-reactive protein) and PCT (procalcitonin) are reliable biomarkers for early detection of AL [21]. It remains a question whether PG-SGA scores, diabetes, and other nutritional indicators can be used as predictors of AL. Although there remains controversy, we believe that early assessment of the nutrition and early intervention for patients with poor nutritional status will reduce the occurrence of AL. At present, intraoperative preventive measures based on patients with high risk of AL are also under study, and great results have been achieved; these include placing a polyurethane vacuum sponge at the anastomosis or blocking drainage on the anastomosis [26, 27], effectively reducing the incidence of AL. Some reasonable clinical strategies can be selected according to the nutritional status of the patient, including increasing abdominal drainage or preventive ostomy [22]. In previous studies, many significant factors, such as AL, were more common in men than in women. But the present study is limited by small sample size, in a single center, with statistical bias and some other aspects. Large-scale, multicenter clinical research is still needed. However, we believe that, with the continuous improvement of surgical techniques and assistive technologies, the incidence of AL will gradually decrease and the number of reoperations due to AL will decline. More importantly, the prognosis of these patients will be improved.

## Figures and Tables

**Table 1:** Analysis of basic clinical information and AL in patients.

Parameters		Anastomotic leak		*p*
		No (326)	Yes (56)	
Sex	Male	199	36	0.645
	Female	127	20	
Age		60.92 ± 10.68	60.59 ± 8.47	0.796
Weight (kg)		64.97 ± 11.15	65.34 ± 12.47	0.820
Height (m)		1.66 ± 0.77	1.68 ± 0.76	0.122
BMI		23.39 ± 3.14	23.03 ± 3.56	0.444

**Table 2:** Single factor analysis of postoperative AL and clinical parameters of patients.

Parameters		Anastomotic leak		*p*
		No (326)	Yes (56)	
NRS-2002	<3	301	40	<0.001
	≥3	25	16	
PG-SGA	0-3	254	6	<0.001
	4-8	71	37	
	>8	1	13	
History of smoking	No	258	40	0.198
	Yes	68	16	
History of alcohol drinking	No	279	46	0.504
	Yes	47	10	
Hypertension	No	247	45	0.455
	Yes	79	11	
Coronary heart disease	No	287	48	0.625
	Yes	39	8	
Diabetes	No	293	32	<0.001
	Yes	33	24	
History of abdominal surgery	No	275	50	0.305
	Yes	51	6	
Gut preparation	Traditional	93	16	0.274
	Laxative	189	28	
	Both	44	12	
Incomplete intestinal obstruction	No	310	52	0.488
	Yes	16	4	
Perioperative blood transfusion	No	322	36	<0.001
	Yes	4	20	
Diarrhea	No	273	17	<0.001
	Yes	53	39	
T stage	1	10	0	<0.001
	2	85	1	
	3	154	30	
	4	77	25	
N stage	0	195	19	<0.001
	1	68	15	
	2	63	22	
M stage	0	294	25	<0.001
	1	32	31	
AJCC	1	78	6	<0.001
	2	116	12	
	3	105	20	
	4	27	18	
Surgical approach	Laparoscopic	57	15	0.096
	Open	265	39	
	Transfer to laparotomy	4	2	
ASA score	1	92	5	0.003
	2	195	38	
	3	39	13	
Neoadjuvant therapy	No	314	55	0.522
	Yes	12	1	

**Table 3:** Single factor analysis of postoperative AL and clinical parameters of patients.

Parameters	Anastomotic leak (*x* + *s*)		*p*
	No (326)	Yes (56)	
Preoperative albumin (g/l)	39.425 ± 3.611	39.016 ± 3.588	0.434
Postoperative albumin (g/l)	31.198 ± 4.252	29.907 ± 5.478	0.046
Preoperative prealbumin (mg/dl)	21.264 ± 5.605	21.941 ± 5.967	0.409
Postoperative prealbumin (mg/dl)	13.907 ± 4.696	13.202 ± 4.670	0.300
Preoperative total albumin (g/l)	65.50 ± 5.105	65.34 ± 4.738	0.826
Postoperative total albumin (g/l)	54.631 ± 6.367	53.704 ± 8.342	0.338
Preoperative hemoglobin (g/l)	134.16 ± 18.601	132.11 ± 20.567	0.453
Postoperative hemoglobin (g/l)	119.38 ± 16.154	118.11 ± 16.200	0.586
Tumor distance from anal margin (cm)	9.184 ± 3.259	7.589 ± 1.727	<0.001

**Table 4:** Multivariate logistic regression analysis of related factors of AL.

Parameter	95% CI		OR	*p*
Postoperative albumin	1.004	1.287	1.137	0.044
Tumor distance from anal margin	0.535	0.886	0.689	0.004
Diabetes	2.063	30.322	7.909	0.003
Perioperative blood transfusion	7.478	436.609	57.139	<0.001
Diarrhea	1.639	16.099	5.136	0.005
T stage	1.983	14.663	5.392	0.001
M stage	3.141	34.088	10.348	<0.001
PG-SGA	6.541	80.792	22.988	<0.001

## Data Availability

The statistical data of the article used to support the findings of this study are available from the corresponding author upon request.
